# Extraction, Characterization, Antitumor and Immunological Activities of Hemicellulose Polysaccharide from *Astragalus radix* Herb Residue

**DOI:** 10.3390/molecules24203644

**Published:** 2019-10-09

**Authors:** Ke Li, Shuying Li, Di Wang, Xiaoxia Li, Xingkang Wu, Xiaojie Liu, Guanhua Du, Xianrong Li, Xuemei Qin, Yuguang Du

**Affiliations:** 1 Modern Research Center for Traditional Chinese Medicine, Shanxi University, Taiyuan 030006, China; like@sxu.edu.cn (K.L.); wuxingkang@sxu.edu.cn (X.W.); liuxiaojie@sxu.edu.cn (X.L.); 2 Key Laboratory of Chemical Biology and Molecular Engineering Ministry, Shanxi University, Taiyuan 030006, China; 3 College of Chemistry and Chemical Engineering, Shanxi University, Taiyuan 030006, China; 4 Shanxi Fruit Industry Work Station, Taiyuan 030001, China; 5 Institute of Materia Medica, Chinese Academy of Medical Sciences, Beijing 100050, China; dugh@imm.ac.cn; 6 Shanxi Jianshuo Food and Drug Research Institute Co. Ltd., Taiyuan 030000, China; XRLi-01@163.com; 7 Shanxi Academy of Traditional Chinese Medicine, Taiyuan 030000, China; 8 Institute of process engineering, Chinese Academy of Sciences, Beijing 100190, China

**Keywords:** *Astragalus radix* herb residue, hemicellulose polysaccharide, characterization, antitumor, immunomodulatory

## Abstract

*Astragalus radix (radix)* have been frequently used for clinical application in China, and the herb residues of *radix* turn out to be a waste of resources. To escape from this, the medicine value of *radix* herb residues is mined in this article. We isolated hemicellulose polysaccharide AX-I-3b from *radix* herb residues by fractional extraction. Monosaccharide-composition analysis revealed that AX-I-3b consisted of arabinose, xylose, and glucose with a molar ratio of 10.4:79.3:1.1. Methylation, NMR and FT-IR analyses showed that AX-I-3b monosaccharide residue was linked as follows: →2,3,4)-β-d-Xylp-(1→, →4)-β-d-Arap-(1→, →4)-β-d-Glcp-(1→. Then, we found that AX-I-3b exhibited antitumor activity against lung cancer in vitro and vivo through MTT assay and xenograft tumor model. Mechanistically, AX-I-3b induced apoptosis in lung cancer cells and xenograft tumors, which is evidenced by the up-regulation of p53, Bax and cleaved caspase-3, and the down-regulation of Bcl-2. Moreover, AX-I-3b synergistically improved the therapeutic ability of cisplatin in xenograft tumors model. Furthermore, AX-I-3b treatment effectively improved the immune organ index, the percentage of spleen lymphocyte subsets and serum cytokine levels in lung cancer mice, supporting that AX-I-3b showed immunomodulatory activity. In conclusion, our results identified AX-I-3b as an antitumor and immunomodulatory agent, providing a new insight into the reutilization of *radix* herb residue.

## 1. Introduction

With China’s high regard for Chinese medicines, the Chinese medicine industry has developed rapidly. Chinese herbal medicines and Chinese patent medicines have been widely accepted and used as the main drugs. However, during the process, large amounts of wastes from Chinese medicines has been produced every day. It is reported that the annual amount of drug residues in China was more than 30 million tons [1]. Chinese medicine slag usually contains high moisture and certain nutrients, which is highly prone to be corrupted, consequently causing serious pollution to the environment. At present, some Chinese medicine slags have been recycled with being used as edible fungi cultivation bases, organic fertilizers, livestock and poultry feed, papermaking, etc. However, the additional values of these products is still low. Additionally, these products may cause secondary pollution to the environment [2]. Therefore, it is of great significance to develop high value-added products of Chinese medicine slag, to establish a recycling industrial chain, to accomplish the benign recycling of ecological resources, to realize the sustainable development strategy of resources, and to solve the serious pollution problems.

*Astragalus radix* (*radix*), a top grade herb, was first recorded in “Shen Nong’s Herbal Classic”. Its medicinal history has been recorded for more than 2000 years. In clinical application, 80% prescriptions of Chinese herbal medicines contain *A. radix*. Due to its importance, *A. radix* has been listed as the 60 strategic key varieties of the state and the 18 major varieties of Chinese medicinal materials in the Ministry of Commerce. Moreover, *A. radix* has been included in the list of national drug and food homology in 2018. At present, the annual production of residues of *A*. *radix* in China exceeds tens of thousands of tons, which is still increasing year by year. Unlike the main components of cell wall hemicellulose of other higher plants, i.e., xyloglucan (no pharmacological activity), our previous studies [3] showed that the main component of the cell wall hemicellulose of *radix* herb residues is arabinoxylan. The main chain and the side chain of arabinoxylan (molecular weight distribution 3.323 × 10^5^–7.256 × 10^6^ Da) is composed of β-1,4-d-xylopyranose and α-l-arabinofuranose, respectively. Current studies have shown [4] that arabinoxylan extracted from wheat bran has anti-tumor activity with significantly inhibiting the growth of tumor-bearing mice and the growth of S180 mouse transplantable tumors, as well as with remarkably promoting thymus and spleen indexes, splenocyte proliferation, natural killer cell and macrophage phagocytosis activity, interleukin 2 production, and delayed-type hypersensitivity reaction.

The amount of arabinoxylan accounts for about 10% of herb residues of *A. radix*, which is 1000 times higher than that of soluble arabinogalactan in the *radix* cytoplasm. The latter has been developed as *A. radix* polysaccharide for injection, which is a commercial drug. However, the extraction process of soluble arabinogalactan consumes a large number of crude herb materials and is costly. At present, the drug is mainly used in leukopenia, lung cancer and other diseases. Yet, it is still not clear whether arabinoxylan of *A. radix* herb residues has anti-lung cancer activity. Therefore, herein, we aimed to explore the mechanisms of immune regulation and promotion of tumor cell apoptosis of arabinoxylan by investigating its chemical structure including monosaccharide composition, junction site, etc. and its antitumor activity both in vitro and in vivo. We aimed to develop high value-added anti-tumor products of arabinoxylan, and to establish a recycling industry chain, as well as to lay a foundation for the benign recycling of ecological resources. Eventually, the findings of this study would contribute to the solution of the serious environmental pollution problems currently faced.

## 2. Results

### 2.1. Isolation and Purification of Ax-I-3

**Figure 1:**
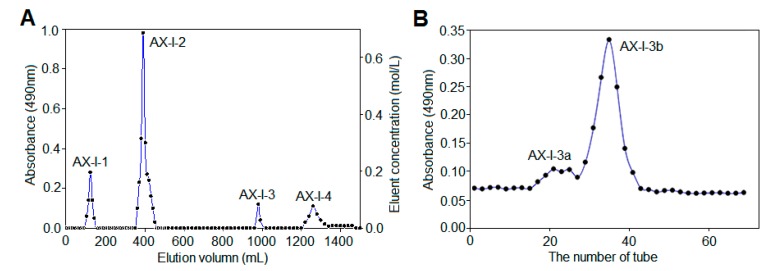
Chromatography of AX-I-3b on DEAE-cellulose 52 Column (**A**) and on SepHacryl S-400 HR gel column (**B**).

Based on the phenol-sulfuric acid method, the elution curve (Figure 1A) indicated that AX-I was separated by DEAE-cellulose 52 to obtain four components with good resolution: water-eluting components AX-I-1, 0.3 mol/L NaCl elution fraction AX-I-2, 0.1 mol/L NaOH elution fraction AX-I-3, and 0.3 mol/L NaOH elution fraction AX-I-4. The polysaccharide was white powder after vacuum freeze drying. As AX-I-1 and AX-I-2 have been studied in our previous work, the present work focused on the components of AX-I-3 fraction. Further purification was carried out using a Sephacryl S-400 HR gel column to obtain a purified AX-I-3b fraction (Figure 1B).

### 2.2. Chemical Properties of AX-I-3b

**Figure 2:**
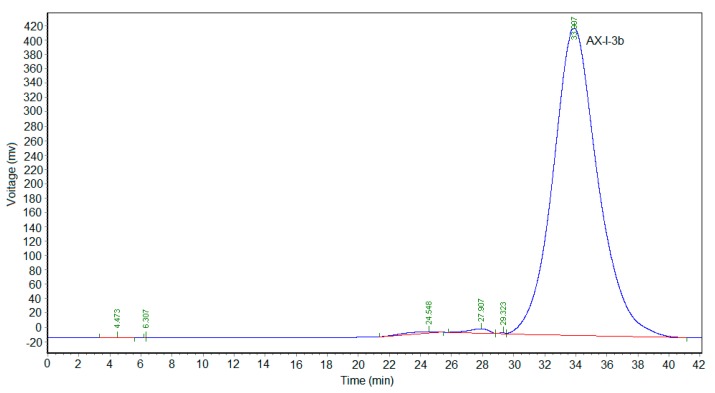
High-performance gel chromatogram of AX-I-3b. The monosaccharide composition of AX-I-3b was analyzed by GC-MS. The total-ion current diagram is shown in Figure 3. Comparing to the retention times of the monosaccharide references, the polysaccharide AX-I-3b contained arabinose (Ara; retention time t_R_ = 25.51 min), xylose (Xyl; t_R_ = 26.25 min), and glucose (Glc; t_R_ = 33.41 min), at a molar ratio of 10.4:79.3:1.1 (Supplementary Table S2).

Dextran was used as the standard. A series of dextran standards with known relative molecular mass was determined using a TSKgel G4000PWxl (10) 7.8 × 300 gel-permeation chromatography column, recording their retention times. Results are shown in Supplementary Table S1. The retention times (t_R_) of dextran were plotted on the horizontal axis and lgMw was plotted on the vertical axis to obtain a standard curve Y = - 0.222975X + 11.4564 (R^2^ = 0.998) (Supplementary Figure S1). The linear relationship was good. The retention time of AX-I-3b was 33.907 min (Figure 2), and the relative molecular mass of AX-I-3b was calculated to be 7.87 kDa.

**Figure 4:**
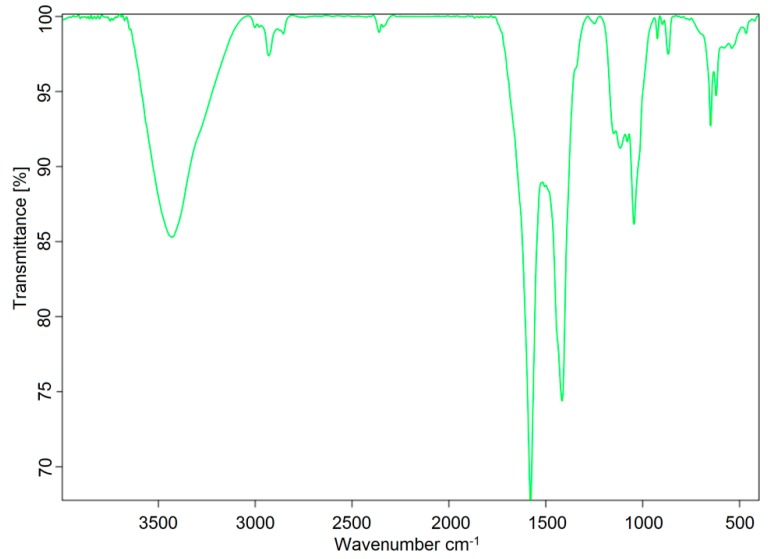
FT-IR spectrum of AX-I-3b.

The FT-IR spectrum showed a strong absorption peak at 3407 cm^−1^ due to the stretching vibration of the chemical bond (O-H) between the oxygen and hydrogen atoms in the hydroxyl group [5]. The absorption peak at 2930 and 1426 cm^-1^ was formed by the expansion and contraction of the chemical bond (C-H) between carbon and hydrogen in the glycosyl group [6]. The absorption peaks at 1119 and 1045 cm^-1^ were formed from the vibration of a chemical bond (C-O-C) between carbon and oxygen in the pyranose ring [7]. The peak at 892 cm^-1^ was the absorption of the formation of the β-pyranose ring [8]. According to the above analysis, the main chain of AX-I-3b was a β-pyran-type polysaccharide. The FT-IR spectrum is shown in Figure 4.

**Figure 5:**
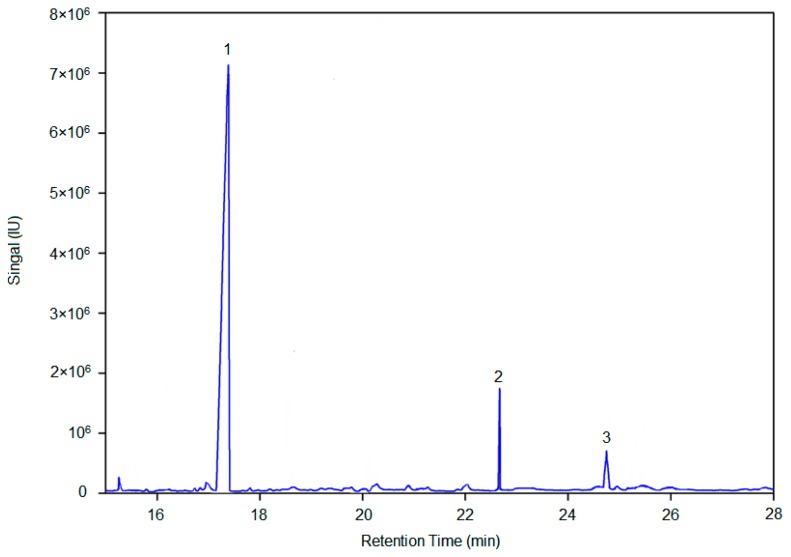
GC-MS total-ion chromatogram of AX-I-3b methylation.

AX-I-3b was methylated by CH_3_I, hydrolyzed by trifluoroacetic acid, reduced by NaBH_4_, and then acetylated by acetic anhydride. The methylated alditol acetates of AX-I-3b were dissolved in CH_2_Cl_2_ and analyzed by GC/MS. Figure 5 shows the GC-MS total-ion chromatogram of methylation analysis of AX-I-3b. By comparing the retention time and characteristic of ion fragments of methylated polysaccharide with the CCRC database (https://www.ccrc.uga.edu/specdb/ms/pmaa/pframe.html), the linkage patterns of AX-I-3b were assigned (Table 1). The following conclusion can be drawn from Table 1: (1) Xylcose residues were presented as 1,2,3,4-linked Xylp residues; (2) Arabinose residues were presented as 1,4-linked Arap residues; (3) Glucose residues were presented as 1,4-linked Glcp residues.

**Figure 6:**
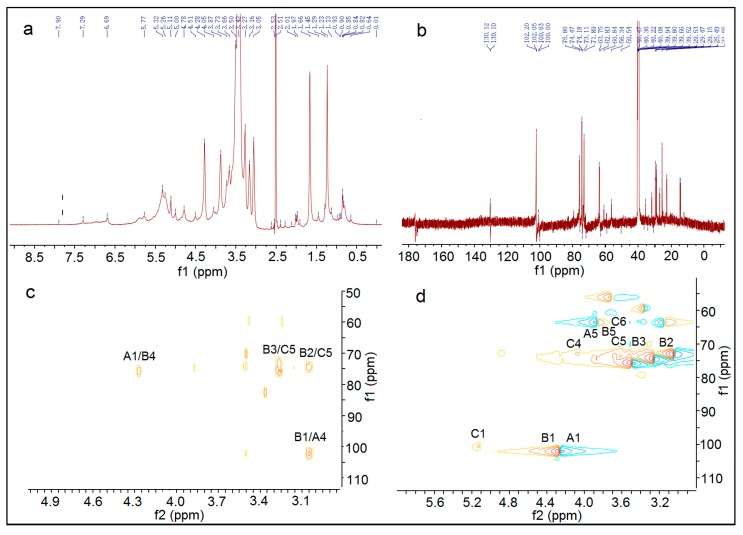
^1^H-NMR (**A**), ^13^C-NMR (**B**), HMBC (**C**), and HSQC (**D**) Spectra of AX-I-3b.

The ^1^H NMR, ^13^C NMR, HMBC, and HSQC spectra of AX-I-3b are shown in Figure 6. The signals of AX-I-3b in 1D NMR (^1^H NMR, ^13^C NMR) and ^2^D NMR (HMQC, HMBC) were analyzed based on the chemical shift of the glycosyl group. According to the chemical shift of this group in literature [9], the signals were assigned separately. The glycosyl groups in AX-I-3b were labeled separately as →4)-*β*-d-Arap-(1→ labeled as A, →2,3,4)-*β*-d-Xylp-(1→ labeled B, →4)-*β*-d-Glcp -(1→ is labeled as C. The different hydrocarbon chemical shifts in each of the AX-I-3b glycosyl groups are shown in Table 2.

The above results of glycosyl composition and the correlation peaks in the HMBC and HSQC maps were used to analyze the linkage sequence of glycosyl groups. In the HMBC spectrum of AX-I-3b, the correlation peak δ4.27/75.2 can be seen; δ4.27 was closest to δ4.28 of 1-H of A, and δ75.2 was closest to δ75.9 of C4 of B, which suggested that A may be linked to B through a (1→4) glycosidic bond. In the correlation peak δ3.04/102.1, δ102.1 was closest to δ102.0 of C1 of B, and δ3.04 was closest to δ3.05 of 4-H of A, suggesting that B may be linked to A through a (1→4) glycosidic bond. Among the correlation peaks δ3.75/100.5, δ3.25 was closest to δ3.27 of 2-H of B, and δ100.5 was closest to δ100.6 of C1, indicating that C may be linked to B through a (1→2) glycosidic bond. Based on the above results, AX-I-3b may be alternately joined by Ara and Xyl through a (1→4) glycosidic bond to form the main chain, and Glc and Xyl may be linked by a (1→2) glycosidic bond to form a branch.

### 2.3. AX-I-3b Inhibits the Growth of Transplanted Tumor in Lewis Lung Cancer Mice

Since many articles have shown that the polysaccharides from *A. radix* exhibit anti-tumor activity [10,11,12,13], it is of interest to know whether AX-I-3b does this or not. To test this, the in vivo anti-tumor activity of AX-I-3b was evaluated on Lewis lung cancer mice. It was observed that mice in the DDP (cisplatin, a chemotherapeutic drug) group had a poor mental state, the mouse fur was obviously rough, the feeding ability was reduced, and the activity was limited. We did not observe obvious changes in other groups.

**Figure 7:**
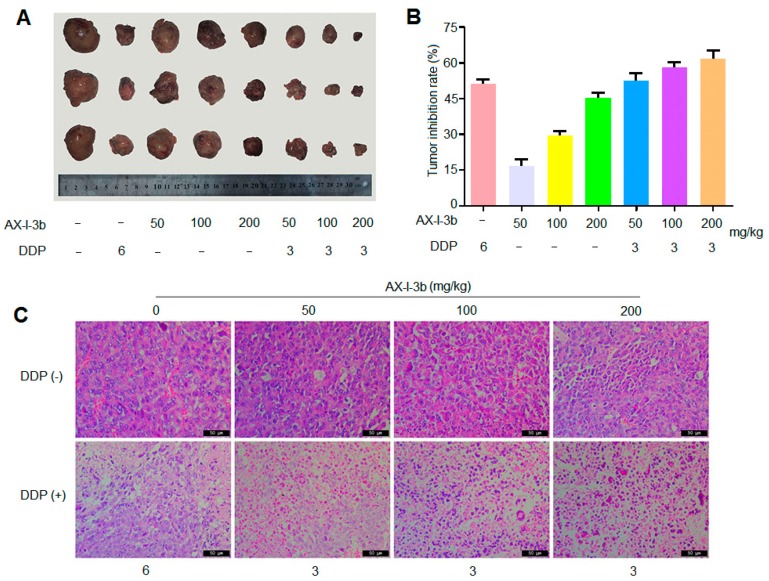
AX-I-3b inhibits tumor growth of Lewis lung cancer in mice. (**A**) The transplanted tumors of Lewis lung cancer mice treated with indicated compounds; (**B**) The growth inhibition rate of indicated compounds against transplanted tumors; (**C**) Tumor pathological section of transplanted tumors (stained with HE, ×400). Scale bars, 50 μm.

As shown in Figure 7A,B, compared with the model group, the tumors of the transplanted tumor mice in AX-I-3b group at the middle and high doses, as well as in the combined drug group, were significantly smaller (*p* < 0.05). Compared with the DDP alone group, the tumor inhibition rate of DDP combined with medium and high dose AX-I-3b administration group was significantly increased with 58% and 61%, respectively, and the differences were statistically significant.

Paraffin section staining is shown in Figure 7C. The tumor cells in the model group were densely arranged, and cells were not deformed. The shape was spherical, the volume was not equal, and the dispersion was diffuse and the staining was deep. In the DDP group, the tumor cells were more severely deformed, the volume was reduced, and the nucleus was stained lightly. The number of necrotic cells was observed. The growth of tumor cells was obviously inhibited. The cell morphology of the polysaccharide alone group was less changed. Tumor cells in the combination group showed large necrosis and nuclear fragmentation, and pyknosis, indicating that cell growth was inhibited to some extent. In conclusion, we found that AX-I-3b is a potent antitumor agent. AX-I-3b alone, or in combination with DDP, significantly inhibits growth of tumor.

### 2.4. AX-I-3b Improves the Immunity of Lewis Lung Cancer Mice

**Figure 8:**
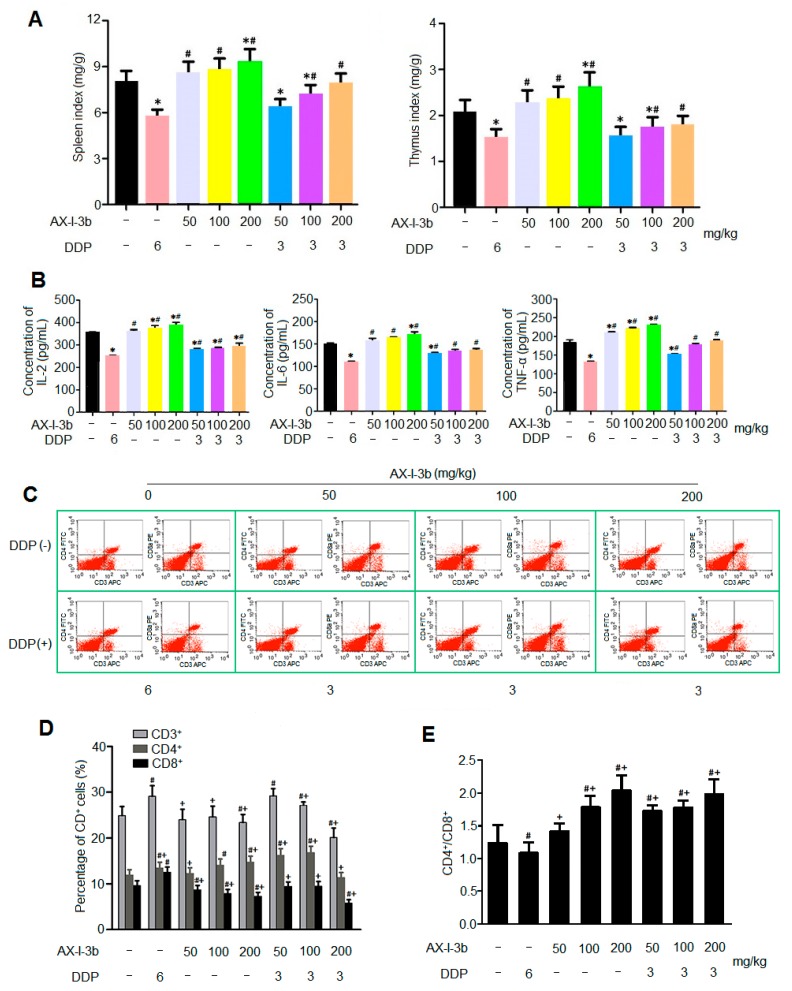
AX-I-3b improved immunity of Lewis lung cancer mice. (**A**) AX-I-3b increased immune organ index in Lewis lung cancer mice; (**B**) AX-I-3b increased serum cytokines levels in Lewis lung cancer mice; (**C**) Distribution of T cell subsets in the spleen of Lewis lung cancer mice by flow cytometry; (**D**) The effect of AX-I-3b on the distribution of spleen T cell subsets; (E) The effect of AX-I-3b on the CD4^+^/CD8^+^. * *p* < 0.05 compared with groups without drug treatment; ^#^ *p* < 0.05 compared with groups treated with DDP (6 mg/mL).

Polysaccharides from *A. radix* are generally used as an immunomodulatory treatment in the clinic [14], suggesting that AX-I-3b probably regulates immunity. To confirm this, the effect of AX-I-3b on immunity was investigated. As shown in Figure 8A, compared with the model group, the organ index of the spleen and thymus of the DDP alone group was significantly decreased, indicating that the immune organs were damaged after DDP administration, and the immune function was inhibited [15], while the organ index of spleen and thymus of AX-I-3b alone group increased, showing a certain protective effect of polysaccharide on immune organs. Compared with the DDP group, the immune organ indices of the thymus and spleen of the AX-I-3b alone group and the combined group significantly increased. The immune organ index of the combined drug group was higher than that of the DDP group while lower than that of the AX-I-3b group, which may relate to the immune organ damage caused by DDP.

AX-I-3b can increase the levels of cytokines IL-2, IL-6 and TNF-α in the serum of Lewis lung cancer mice. The IL-2 secretion in the middle and high dose AX-I-3b group and the combined drug group was significantly different from that in the model group (*p* < 0.05). Specifically, the AX-I-3b group and the combined drug group and compared with the DDP group, the IL-2 secretion was increased. The amount of IL-6 secreted in the high-dose AX-I-3b group and the combined low-dose group was significantly different from that in the model group. Compared with the DDP group, the amount of IL-6 secretion in AX-I-3b group and the combined drug group increased. The amount of TNF-α secreted by the AX-I-3b group and the combined low dose group was significantly different from that of the model group. Specifically, compared with the DDP group, the level of TNF-α in AX-I-3b group and the combined drug group increased. To sum up, the results showed that AX-I-3b could significantly inhibit the decrease of IL-2, IL-6 and TNF-α in the serum of Lewis lung cancer mice (Figure 8B). As T cell maturation is closely related to the thymus [16], and IL-2, IL-6 and TNF-α are released by activated T cells [17], the levels of the three cytokines are consistent with the trend of immune organ index.

T lymphocytes, important cells that constitute the body’s immune defense system, play an important role in the development process of tumor, treatment and prognosis. CD3^+^, CD4^+^ and CD8^+^ are the clusters of differentiation antigens on the membrane of T lymphocytes, among which CD3^+^ is a unique marker for T cells, CD4^+^ and CD8^+^ are markers for helper T cells and cytotoxic T cells, respectively. Among them, CD4^+^ T lymphocytes can also help B cells produce antibodies, including IgG, IgM and other antibodies [18]. CD8^+^ T lymphocytes mainly have opposite immunosuppressive effects [19]. Therefore, we can effectively understand the occurrence and development of tumors and the anti-tumor effects of drugs by detecting T lymphocytes and their subpopulations. As shown in Figure 8C,D, after the drug intervention, the number of CD4^+^ T cells in the polysaccharide group and the combined group was significantly increased compared with the model group, indicating the proportion of CD4^+^ T cells and CD8^+^ T cells was significantly increased. Compared with the DDP group, the ratio of CD4^+^ T cells and CD8^+^ T cells was significantly increased. It is suggested that AX-I-3b may improve the cellular immune function of mice by increasing the ratio of CD4^+^ T cells and CD8^+^ T cells and can alleviate the immunosuppression caused by DDP administration. All these results suggest that AX-I-3b may inhibit tumor growth through activating the immune system. Altogether, these results show that AX-I-3b improves the immunologic function of lung cancer mice, potentially contributing to its antitumor activity.

### 2.5. AX-I-3b Induces Apoptosis in Tumor Tissues

**Figure 9:**
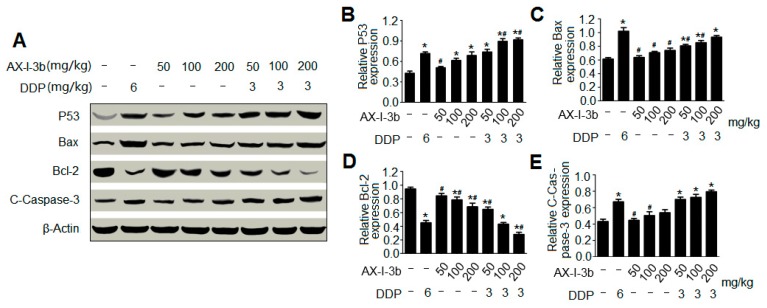
AX-I-3b induced apoptosis in tumor tissues. Data are shown as means ± SD. (n = 3). * *p* < 0.05 compared with the model group; ^#^ *p* < 0.05 compared with with the DDP group. (**A**) Western blot analysis of expression of apoptotic proteins in tumor tissues; (**B**) The expression of p53; (**C**) The expression of Bax; (**D**) The expression of Bcl-2; (**E**) The expression of caspase-3.

To dissect the mechanism by which AX-I-3b inhibits tumor growth, the effects of AX-I-3b on apoptotic proteins were assayed (Figure 9A). The imbalance of cell proliferation and apoptosis is an important feature of the occurrence and development of lung cancer. Various factors resulting in impaired apoptosis can lead to tumor production. Apoptosis is a tightly regulated process involving differences in the expression of various proteins. Among the various factors associated with apoptosis, some pro-apoptotic proteins and anti-apoptotic proteins can serve as markers of apoptosis. The tumor suppressor protein p53, both an inducing factor in apoptosis and a regulator of apoptosis [20] is involved in the regulation of DNA damage repair, cell cycle and apoptosis [21]. The induction of apoptosis is central to the tumor-suppressive activity of p53 [22]. Upon activation by DNA damage-induced or oncogene-induced signaling pathways, p53 promotes the expression of a number of genes that are involved in apoptosis, including those encoding death receptors [23,24] and proapoptotic members of the Bcl-2 family [25,26]. In most cases, p53-induced apoptosis proceeds through the mitochondrial release of cytochrome c, which leads to caspase activation [27]. Western blot was used to detect the expression level of p53 in tumor tissues. The results showed that the expression of p53 was increased in a concentration-dependent manner in the polysaccharide group at all tested doses and in the combination group (Figure 9B). Among them, the expression levels of the combined low dose drug group and the DDP group were comparable, indicating that AX-I-3b induced tumor cell apoptosis was involved in the increasing expression of p53.

A large number of studies has demonstrated that Bcl-2 family proteins are important mediators of apoptosis [28] and play an important role in the mitochondrial apoptotic pathway. Bcl-2 is an anti-apoptotic member of the Bcl-2 family and prevents the release of cytochrome c from mitochondria to cytosol, whereas Bax is an important pro-apoptotic member of the Bcl-2 family that promotes the release of cytochrome c from mitochondria to cytosol [29]. The high expression of Bcl-2 anti-apoptotic protein and the low expression of Bax pro-apoptotic protein can inhibit cell apoptosis, prolong cell survival, and increase cell number, which are one of the important mechanisms of lung cancer. Therefore, the ratio of Bax/Bcl-2 is the main checkpoint of the endogenous apoptotic pathway [30]. Western blot analysis showed that Bcl-2 expression was down-regulated while Bax expression was up-regulated compared with the model group. The polysaccharide group at all tested doses and the combination group significantly increased the ratio of Bax/Bcl-2 and in a dose-dependent manner (Figure 9C,D. AX-I-3b treatment caused mitochondria-dependent apoptotic pathway in mouse tumor cells through up-regulating the Bax/Bcl-2 ratio.

Cytochrome c is released from the mitochondria into the cytosol, which binds to Apaf-1, ATP and procaspase-9 to form the apoptosome assembly [31] and triggers the activation of the caspase-dependent mitochondrial apoptotic pathway [32]. The apoptosome complex activates caspase-9 and ultimately activates the downstream caspase-3 to perform the apoptotic process [33]. In this study, the expression of downstream caspase-3 protein was analyzed by Western blot, and the effect of mitochondria-mediated endogenous apoptosis pathway on apoptosis of mouse tumor cells was explored. As shown in Figure 9E, the expression of caspase-3 was increased in the polysaccharide group at all tested doses and in the combination group, and the expression of the low-dose combination group was similar to that of the DDP group, suggesting that the mitochondria-mediated apoptotic pathway might be involved in AX-I-3b-induced apoptosis in mouse tumor cells.

### 2.6. Effect of AX-I-3b on A549 Cells in Vitro

**Figure 10:**
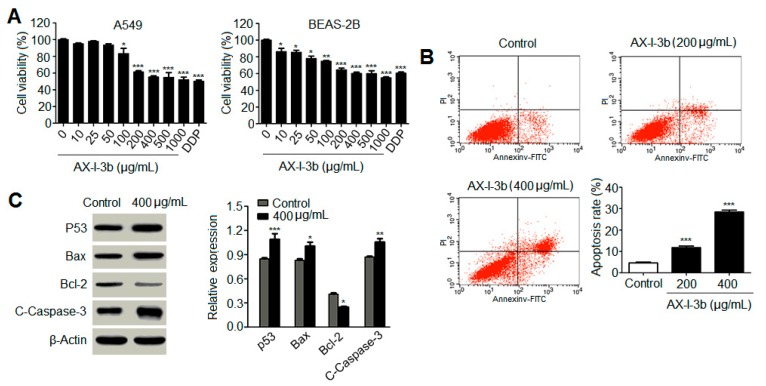
Effect of AX-I-3b on A549 cells. (**A**) Effect of AX-I-3b on the cell viability of A549 cells; (**B**) The apoptosis detection of A549 cells induced by AX-I-3b; (**C**) Effect of AX-I-3b on apoptosis protein and anti-apoptotic protein expression in A549 cells by Western blot analysis. Values are means ± SD (n = 3). * p < 0.05, ** *p* < 0.01 and *** *p* < 0.001 as compared with the control.

To further determine the mechanism that AX-I-3b induces apoptosis in tumor, the effect of AX-I-3b on apoptosis was monitored in A549 and BEAS-2B cell lines. MTT assays were performed at AX-I-3b concentrations of 0, 10, 25, 50, 100, 200, 400, 500, and 1000 μg/mL. As shown in Figure 10A, AX-I-3b inhibited the proliferation of human lung-adenocarcinoma A549 cells in vitro and also slightly inhibited BEAS-2B human normal-lung epithelial cells. When the concentration of AX-I-3b was between 200 and 1000 μg/mL, a significant difference in cell viability was observed between the treatment group and the blank group. The chemotherapy drug cisplatin was used as a positive control. The AX-I-3b group at the concentration range of 200–500 μg/mL was equivalent to cisplatin for BEAS-2B, while the inhibition effect on A549 cells was slightly poorer than that of cisplatin.

After 24 h of AX-I-3b intervention, the proportion of G1, S, and G2 cells in the 200 and 400 μg/mL groups significantly differed from that in the negative control group. Furthermore, the group at 400 μg/mL was more significant than that at 200 μg/mL. As the drug concentration increased, the G1 phase gradually increased while the proportion of cells in the S and G2 phases decreased. This finding indicated that AX-I-3b intervention can arrest the human lung-adenocarcinoma A549 cells in the G0/G1 phase (Supplementary Figure S2 and Supplementary Table S3).

As shown in Figure 10B and Supplementary Figure S3, the spontaneous apoptosis rate of A549 cells treated with AX-I-3b was 5.31%. When treated with 200 and 400 μg/mL polysaccharide for 24 h, the apoptotic rate of A549 cells was 13.43% ± 2.32% and 28.22% ± 1.96%, respectively. Compared with the negative control group (4.98% ± 0.59%), the apoptotic rate increased with statistically significant difference. Results showed that AX-I-3b significantly promoted A549 cell apoptosis.

Western blotting was used to detect the changes of related proteins in A549 cells treated with AX-I-3b at 400 μg/mL for 24 h. As observed in Figure 10C, after AX-I-3b intervention, the protein expression of p53, Bax and cleaved caspase-3 protein increased whereas the protein expression of Bcl-2 decreased. Compared with the blank group, each relative protein expression has a certain difference. The results indicated that AX-I-3b down-regulated the expression of anti-apoptotic protein Bcl-2 but up-regulated the expression of pro-apoptotic proteins p53, Bax and cleaved caspase-3 to induce apoptosis in A549 cells. As described above, we conclude that AX-I-3b induce apoptosis in lung cancer tissues and cells, establishing basic mechanism of its antitumor activity.

## 3. Conclusions and Discussion

In summary, the hemicellulose polysaccharide was isolated from *Astragalus Radix* herb residue. The physicochemical properties, chemical structure, and antitumor activities of AX-I-3b were studied. Results showed that AX-I-3b was a heteropolysaccharide containing arabinose (t_R_ = 25.51 min), xylose (t_R_ = 26.25 min), and glucose (t_R_ = 33.41 min) at a molar ratio of 10.4:79.3:1.1. The main chain of this component was a β-pyran type polysaccharide, and the connection manner of the monosaccharide residue was →4)-β-d-Arap-(1→, →2,3,4)-β-d-Xylp-(1→, →4)-β-d-Glcp-(1→.

Currently, chemotherapy remains the main treatment option for cancer patients. However, the severe clinical side effects limit its therapeutic use [34,35]. In this study, the antitumor activity in vitro was studied. Results showed that AX-I-3b significantly inhibited the proliferation of human lung-adenocarcinoma A549 cells and induced apoptosis. In vivo anti-tumor experiments demonstrated that intraperitoneal injection of AX-I-3b significantly delayed the growth of transplanted tumors in Lewis lung cancer mice. DDP alone can inhibit the growth of transplanted tumors in Lewis lung cancer mice and high doses of AX-I-3b can greatly enhance this effect. On the other hand, DDP reduced the immune function of Lewis lung cancer mice, but this inhibition was largely offset by AX-I-3b.

In the blood, since most of the tumor cells can be cleared by the host’s immune surveillance function, only a very small number of tumor cells can form distant metastases. Therefore, tumor patients can only gradually recover if they regulate and improve immune function [36]. Modern medicine also believes that the so-called "carcinogenic factors" are actually only a condition for tumorigenesis. The key factor for tumorigenesis and development is the low function of the immune system, and this view coincides with the theory of "positive virtual tumor" of Chinese medicine [37]. It has been confirmed that during the four processes of tumor, i.e., occurrence, development, invasion and metastasis, many Chinese medicines can achieve the effect of inhibiting tumor by enhancing the body’s immune function [38,39]. T lymphocyte-mediated cellular immunity is the main factor in tumor immunity. Therefore, the level of T lymphocyte transformation rate (proliferation index) can be used as one of the indicators reflecting the immune function of the body. In the tumor-bearing state, the host’s T cells have changed in terms of number, function, and proportion of subpopulations. In the T lymphocyte subset, CD4^+^ represents helper T cells (Th), which secrete IL-2 and IFN-γ, and cooperate with natural killer cells (NK) to kill tumor cells and assist immune response. CD8^+^ represents inhibition T cells (Ts), which can inhibit the immune response of cells by inhibiting the helper function of Th cells, and can directly inhibit humoral immunity, which is opposite to that of CD4^+^ cells. Regulatory T cells (Tregs) are a subset of T cells that have a negative regulatory role in suppressing immune function. Peripheral CD4^+^ T cells express the IL-2 receptor alpha chain CD25, which lacks an autoimmune disease, CD4^+^CD25^+^ regulatory T cell Treg, an immunological marker that regulates its development and function [40], therefore the number of Tregs is closely related to the prognosis of the tumor [41].

Studies have shown that [42] the use of formula containing *Astragalus radix* can significantly increase the thymus and spleen index of tumor-bearing mice, increase the percentage of CD4, CD3, CD19 and CD8 lymphocytes, thereby inhibiting lung metastasis. In this study, the results of spleen T lymphocyte assay showed that AX-I-3b may improve the cellular immune function of mice by increasing the ratio of CD4^+^ T cells and CD8^+^ T cells.

Western blot analysis showed that AX-I-3b treatment significant increased expression of p53 protein, increased expression of proapoptotic protein Bax and decreased the expression of anti-apoptotic protein Bcl-2. Cytochrome c was released from mitochondria into the cytosol, and then the expression of caspase-3 protein increased. Thus, this induces tumor cell apoptosis. In combination with a half dose of DDP, these effects can enhance the chemotherapeutic effect and reduce the toxicity of DDP.

**Figure 11:**
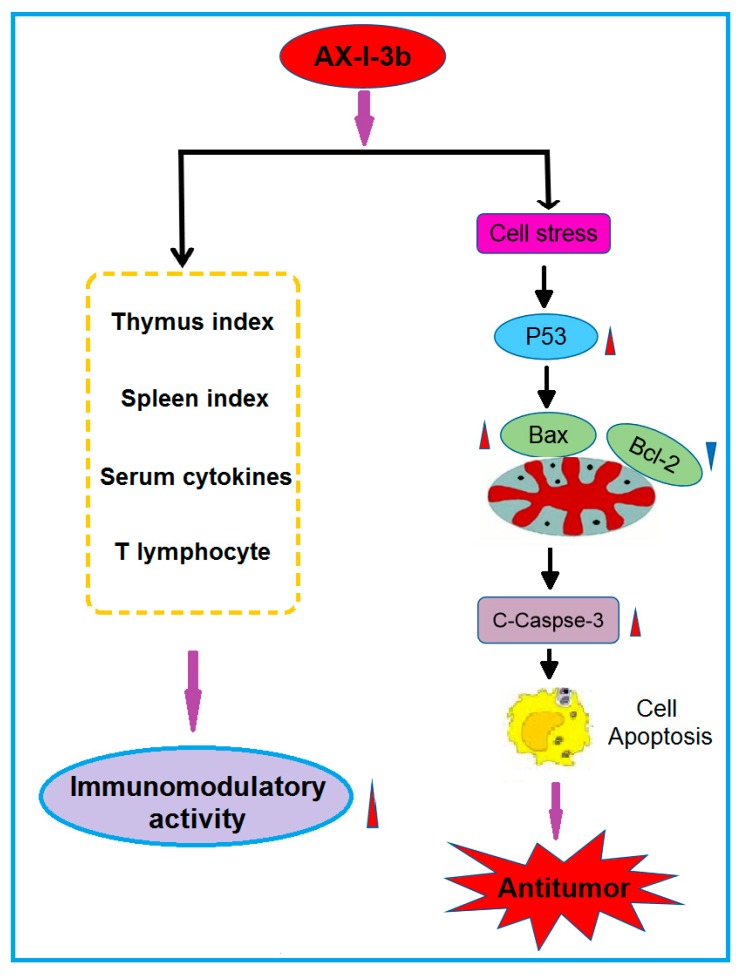
Immunomodulatory activity and potential anti-tumor mechanism of AX-I-3b.

Collectively, the present findings suggest that AX-I-3b can enhance the body’s immunity and induce apoptosis through the caspase-3 dependent mitochondrial pathway, which has potential for chemoprevention and adjuvant therapy of lung cancer. Possible immune and antitumor mechanisms are shown in Figure 11. Although the immunomodulatory and antitumor mechanisms of AX-I-3b remain to be further exploited, this research has reference value for the development of additional products of AR herb residues. The use of active ingredients in AR herb residue can not only reduce the consumption of AR resources, but also reduce pollution.

## 4. Materials and Methods

### 4.1. Materials and Chemicals

*Astragalus radix* was obtained from the wild *A. membranaceus* (Fisch.) Bge.var.mongholicus (Bge.) Hsiao in Hunyuan (Shanxi, China). It was identified as a dry root of the leguminous plant *A. membranaceus* (Fisch.) Bge. Var. mongholicus (Bge.) Hsiao by Professor Qin Xuemei of Shanxi University. The specimens were stored at the Modern Research Center of Traditional Chinese Medicine of Shanxi University. Diethylaminoethyl (DEAE)-cellulose 52 and Sephacryl S-400 HR were purchased from Beijing Solarbio Science & Technology Co., Ltd (Beijing, China). Anhydrous dimethyl sulfoxide (99.7% pure; passed through a molecular sieve) was purchased from the Beijing Bailingwei Technology Co., Ltd (Beijing, China). Sodium borohydride (NaBD_4_) and Deuterated dimethyl sulfoxide were bought from Shanghai Jianglai Biotechnology Co., Ltd (Shanghai, China). The dextran standards including Dextran 4320, Dextran 12600, Dextran 73800, Dextran 110000, Dextran 289000, and Dextran 496000 were obtained from China Institute of Metrology (Beijing, China). Ribitol, fucitol, arabitol, rhamnitol, mannitol, xylitol, sorbitol, and galactitol were bought from Sigma (St. Louis, MO, USA). All other reagents were of analytical grade. Cisplatin and double antibody (Hyclone penicillin-streptomycin) were purchased from Sigma. Phosphate-buffered saline (PBS), DMEM high-glucose medium, trypsin, fetal bovine serum (FBS), MTT reagent, and dimethylsulfoxide (DMSO) were purchased from Beijing Suo Laibao Technology Co., Ltd (Beijing, China). Antibodies against p53, Bcl-2, Bax, caspase-3, β actin and ELISA detection kits (IL-2, IL-6 and TNF-α) were purchased from the Wuhan Boster Bioengineering Co., Ltd (Wuhan, China). 

### 4.2. Polysaccharide Extraction

Preparation of *A. radix* residue: *A. radix* material was pulverized and passed through a 100-mesh sieve. Then, 20 g powder was weighed into a 1000 mL flask to which 600 mL of water was added. After the mixture was heated in an electric heating jacket at 42 °C for 1 h and centrifuged (3000 r/min, 10 min), the supernatant was discarded. This extraction process was repeated for three times, and the resulting precipitate was dried at 30 °C to obtain a Scutellaria residue.

Pretreatment of *A. radix* residue: To prepare alcohol-insoluble residue (AIR) powder, 20 g *A. radix* residue powder was weighed according to a previous method [43], and 600 mL of 80% ethanol solution was added. After the mixture was heated in a water bath at 60 °C for 1 h and centrifuged (3000 r/min, 10 min), the supernatant was discarded. The process was repeated for several times until the upper layer was clear and colorless. After centrifugation for 10 minutes at 3000 r/min, the precipitate was collected. The acetone solution was added to the above precipitate at a solid-to-liquid ratio of 1:10. Afterwards, the mixture was allowed to stand. After discarding the supernatant, the precipitate was dried in an oven at 45 °C. Porcine pancreatic α-amylase solution was added to the dried powder (material-to-liquid ratio = 1:100; 20 U/mL; concentration = 50 mmol/ L Tris-HCl buffer; pH 7.0). The mixture was then incubated for 12 h at 37 °C in a 200 r/min shaker and centrifuged (3000 r/min, 10 min). The precipitate was washed repeatedly with distilled water adding with acetone solution (feed ratio = 1:10), and then was allowed to stand still. After centrifugation at 3000 r/min for 5 min, the supernatant was discarded. The precipitate was dried in an oven at 45 °C to obtain AIR powder.

Extraction of hemicellulose (AX) from *A. radix* residue: According to a previous method [44], 20 g AIR powder was placed in a 1000 mL Erlenmeyer flask, adding 400 mL of sodium chlorite solution with a mass fraction of 0.6%, of which the pH was adjusted to 4.2 to 4.7 with glacial acetic acid. The flask was sealed and placed in a water bath at 75 °C, with shaking manually every 20 min. Sodium chlorate solution (0.6%) was added after 1 h. The pH was adjusted to 4.2–4.7 by adding glacial acetic acid. The above process was repeated for 1 h. After the completion of the reaction, the bottle wall was immediately rinsed with cold water to room temperature and centrifuged (3000 r/min, 10 min). And then the precipitate was collected. The precipitate was repeatedly washed with distilled water to neutrality, centrifuged at 3000 r/min for 10 min, and dried in an oven at 50 °C to obtain hemicellulose.

According to the previous method [45], dried hemicellulose (10 g) was put in a 1000 mL Erlenmeyer flask, to which 500 mL pectinase (3 U/mL and sodium acetate 50 mmol/L; pH 5.0) was added. After being incubated at 24 °C in a 200 r/min shaker for 24 h, the mixture was centrifuged (3000 r/min, 10 min), and then the supernatant was discarded. This procedure was repeated for three times, and the pellet was washed for three times with 500 mL of distilled water. After centrifugation, 500 mL of a mixed solution of Na_2_CO_3_ (50 mmol/L) and EGTA (mmol/L) was added to the precipitate. The mixture was incubated at 24 °C in a 200 r/min shaker for 24 h and centrifuged, and then the supernatant was discarded. This step was repeated for three times, and the precipitate was washed three times with 500 mL of distilled water.

A 500 mL mixed solution of 1 mol/L KOH and 1% NaBH_4_ (liquid-to-liquid ratio = 1:50) was added to the obtained precipitate. The mixture was incubated for 12 h at 24 °C in a 200 r/min shaker and centrifuged. The supernatant was then collected. This step was repeated for three times. Afterwards, supernatant was combined and stored in a refrigerator at 4 °C to obtain the hemicellulose component I. To adjust the pH to 5.0, glacial acetic acid at certain amount was added to the above hemicellulose component solution. And then, dialysis against distilled water was performed four times for 12 h each time. The product was concentrated and lyophilized to obtain polysaccharide of hemicellulose component I of *A. radix* residue, labeled as AX-I.

### 4.3. Sevage Method for Protein Removal

The protein in AX-I was removed by using a previous method [46]. A certain amount of *A. radix* residue polysaccharide AX-I was weighed and reconstituted with water in a separatory funnel, after which one-fourth volume of Sevage reagent (CHCl_3_:CH_3_(CH_2_)_3_OH = 4:1) was added. The funnel was shaken for 15 min. After standing, the lower and middle layers were discarded. The Sevage reagent was further added to the upper solution and shaken for 15 min. This step was repeated for three to four times until the protein layer disappeared.

### 4.4. Separation and Purification of Hemicellulose Polysaccharide

According to a previous method [47], 0.2 g polysaccharide powder was weighed and dissolved in 2 mL distilled water for DEAE-cellulose 52 column chromatography. Each tube was collected every 10 min at a flow rate of 0.6 mL/min. First, it was eluted with distilled water until no sugar was detected. And then, it was eluted with 0.3 mol/L NaCl solution and 0.6 mol/L NaCl aqueous solution. Finally, it was eluted with 0.1 mol/L NaOH aqueous solution and 0.3 mol/L NaOH aqueous solution. The absorbance of each tube solution at 490 nm was determined by phenol-sulfuric acid method, according to which the elution curve was drawn. The polysaccharide fractions of each tube were collected according to the curve, concentrated, and dialyzed in a 3 kDa dialysis bag for 48 h, followed by reconcentration and lyophilization to obtain the separated polysaccharides AX-I-1-AX-I-4.

Approximately 0.1 g isolated polysaccharide powder was dissolved in 1 mL of distilled water. Further purification was performed using a Sephacryl S-400 HR gel column. Distilled water was used as the mobile phase at a flow rate of 0.5 mL/min. Each tube was collected every 10 min. The phenol-sulfuric acid method was used to detect and collect the polysaccharides of each component and then concentrate and freeze dry them to obtain a purified hemicellulose component.

### 4.5. Homogeneity and Molecular Weight

The homogeneity and average molecular weight of AX-I-3b were identified by high-performance gel-permeation chromatography (HPGPC). We accurately weighed 1 mg each dextran standard to prepare a 2 mg/mL solution. The mobile phase was pure water, the injection volume was 20 μL, and the flow rate was set at 0.3 mL/min. The gel column model was TSKgel G4000 PWxl (10) 7.8 × 300 mm^2^. The column temperature and the detector temperature was set to 35 °C and 34 °C, respectively. The retention time (t_R_) of each standard was plotted on the horizontal axis and lgMw was plotted on the vertical axis. The calibration was obtained and the linear equation was calculated. The polysaccharide AX-I-3b was formulated into a sample solution with a concentration of 2 mg/mL. The relative molecular mass was calculated by taking the measured t_R_ into the equation.

### 4.6. FT-IR Spectroscopy Analysis

Based on the literature [48], 1–2 mg sample was weighed. The IR of the polysaccharide was determined by KBr tableting. The scanning wavelength was ranged from 4000 cm^−1^ to 400 cm^−1^.

### 4.7. Monosaccharide-Composition Analysis

According to the literature [49], 5 mg of polysaccharide AX-I-3b was weighed, 1.5 mL of TFA at a concentration of 2 mol/L was added. Hydrolysis for 3 h in an oil bath at 110 °C was performed after the reaction. After being dried with air, adding 1 mL of methanol, and re-drying in the air, the process was repeated for three times to obtain a complete acid hydrolysis product. This product was dissolved in a ribitol internal standard solution at a concentration of 0.1 mg/mL, and the mass fraction was added. About 2% NaBH_4_ in DMSO was added, and the mixture was incubated at 40 °C in a 200 r/min shaker for 90 min. After the reaction, pH was adjusted to neutral with glacial acetic acid, in which 500 μL acetic anhydride and 50 μL 1-methylimidazole were then added. After reacting at 25 °C for 10 min and cooling to room temperature, the reaction was stopped by adding 5 mL of water, extracted three times with 1 mL of CH_2_Cl_2_, and the organic phase was combined. Extraction with 1 mL of water was performed for three times and retain. The organic phases were combined. The combined organic phase was dried in air, redissolved in CH_2_Cl_2_, and filtered through a 0.22 μm organic filter. Finally, 2 μL filtrate was injected into the GC-MS.

The monosaccharide standard was weighed and dissolved in dimethyl sulfoxide solution with 0.1 mg/mL ribitol as internal standard. Then, 200 μL of acetic anhydride and 50 μL of 1-methylimidazole were added. The mixture was vortex and let stand, react at 25 °C for 10–15 min. Afterwards, 500 μL distilled water was added to terminate the reaction, add 500 μL of two Methyl chloride, vortex, centrifugation, aspirate the lower layer, repeat the extraction twice, combine the organic phase, blow dry with air, reconstitute with methylene chloride, add anhydrous sodium sulfate to remove water, filter. 1 μL of filtrate was injected into GC-MS analysis to obtain gas chromatography-mass spectrometry of monosaccharide standards.

The monosaccharide composition in the sample was determined by comparing the GC-MS spectra of the monosaccharide standards and samples. The molar ratio of each monosaccharide in the sample was determined by calculating the peak area.

Gas chromatographic conditions: Hue chromatography column, DB-5MS capillary column (30 m × 0.5 cm × 0.25 μm); carrier gas, high purity helium (99.99%); injection volume 2 μL; carrier gas flow rate 1.0 mL/min; split ratio 10:1; inlet temperature 220 °C; temperature programming conditions, the starting temperature is 100 °C; rise to 180 °C at 5 °C /min for 1 min; 1 °C/min to 190 °C for 2 min; to 30 °C/min rises to 220 °C for 2 min; rises to 230 °C at 1 °C/min for 2 min; rises to 280 °C at 20 °C/min for 10 min.

Mass spectrometry conditions: Electron bombardment source (EI); ion source temperature, 220 °C; ion source, 70 eV; transmission line temperature, 250 °C; scan mode for full scan (Full Scan); mass scan range m/z: 30~550.

### 4.8. Methylation Analysis

According to the literature [50], 2 mL of anhydrous DMSO was added to 5 mg polysaccharide AX-I-3b. Ultrasonication was performed in an argon atmosphere until completely dissolution. Then, 50 mg of NaH powder was weighed and placed in a flask, 1 mL of anhydrous DMSO was added, and argon was charged. At 60 °C and 500 r/min, magnetic stirring was performed for 1 h until no hydrogen was produced. The above two liquids were combined and ultrasonically reacted for 1 h under argon-filled conditions. After cooling in an ice bath, adding 1 mL of CH_3_I in the dark, protecting with argon, and ultrasonicating at 20 °C for 30 min three times, 5 mL of 4 mM Na_2_S_2_O_3_ aqueous solution and 5 mL of CHCl_3_ were added to the reaction solution. The lower layer was extracted and aspirated. This process was repeated for five times. The lower-layer liquid was added with 5 mL of distilled water and extracted, and then the upper-layer liquid was discarded. This process was repeated for five times. Anhydrous Na_2_SO_4_ was added to the chloroform phase, which was filtered and dried with nitrogen. The methylation product was detected by infrared spectroscopy, and no absorption peak was detected at 3400 cm^-1^, indicating the completion of the methylation reaction.

About 2 mL of 2 mol/L TFA was added, and the mixture was hydrolyzed at 120 °C for 90 min after sealing. The mixture was air dried, adding with 1 mL of methanol, and re-dried. The process was repeated for three times to obtain a hydrolyzate. And then, 2 mL of freshly prepared NaBD_4_ aqueous solution with a mass fraction of 2% was added, followed by incubation at 40 °C and 200 r/min for 90 min. After the reaction, pH was adjusted to neutral with glacial acetic acid and the solution was dried with nitrogen. We added 500 μL of acetic anhydride and 50 μL of 1-methylimidazole with setting the reaction temperature at 25 °C for 10 min. 1 mL of water was added to end the reaction, after which extraction with 1 mL of CH_2_Cl_2_ was performed for 3 times. The organic phase was combined and extracted with 1 mL of distilled water for three times. The organic phase was combined again, dried with nitrogen, dissolved in CH_2_Cl_2_. After filtration with a 0.22 μm organic filter, 2 μL of the solution was injected into the GC-MS. The conditions of gas chromatography and mass spectrometry were the same as described in 2.7.

### 4.9. NMR Spectroscopy

According to the literature [51], 30 mg dry polysaccharide sample AX-I-3b was dissolved in 0.5 mL DMSO-d6. ^1^H-NMR, ^13^C-NMR, and ^2^D-NMR (HMBC and HSQC) spectra were obtained at 30 °C.

### 4.10. Animal Experiments

#### 4.10.1. Animals and Experimental Design

In total, 72 male SPF-class C57BL/6 mice were used in the study. Except for 8 mice in the blank control group, the remaining 64 mice were injected with 0.2 mL 1 × 10^7^ cells/mL LLC cells at a distance of 1 cm from the right axilla to establish a subcutaneous xenograft model. When the tumor long diameter (L) and short diameter (W) were both 5 mm, the model was considered to be successful, and the transplanted mice were subsequently grouped (n = 8).

The dosing regimen was set as follows: mice in the blank control group and model control group were intraperitoneally injected with normal saline once a day, 0.3 mL each time; mice in the DDP group (6 mg/kg) were intraperitoneally injected once a week, 0.3 mL each time; the low dose AX-I-3b group, middle dose AX-I-3b group and high dose AX-I-3b group (50, 100, 200) mg/kg, intraperitoneal injection of 0.3 mL per day; low dose combination group, middle dose combination group, high dose combination group: 1/2 DDP + AX-I-3b: (3 + 50, 3 + 100, 3 + 200) mg/kg, of which AX-I-3b was intraperitoneally injected 0.3 mL per day at the above doses. DDP was administered at a dose of 3 mg/kg once a week. For all drugs, the total volume was 0.3 mL. Each group started to take the drug on the first day after the successful modeling, lasting for 15 days.

#### 4.10.2. Tumor Inhibition Rate and Immune Organ Index

24 h after the last administration, all mice were weighted and then sacrificed by cervical dislocation. The tumor, spleen and thymus of the mice were then carefully dissected and weighted. Immune organ indexes of the spleens and thymuses were used to express the effects of AX-I-3b on the immune organs, following the formula [52]:

the organ indexes (%) = average weight of organs/(average body weight) × 100%.

We measured the tumor inhibitory rate using the formula: inhibitory rate (%) = (the tumor weight of the model group − the tumor weight of the treatment group)/the tumor weight of the model group × 100% [53].

#### 4.10.3. Measurement of Cytokines

The serum levels of cytokines were determined by ELISA according to the manufacturer’s instructions. ELISA kits were employed to measure the levels of interleukin-2 (IL-2), interleukin-6 (IL-6), tumor necrosis factor-α (TNF-α).

#### 4.10.4. Determination of Spleen T Lymphocyte Subsets

We analyzed the T lymphocyte subsets in the spleen using flow cytometry. The mice were sacrificed by cervical dislocation. And then, the spleen was weighted and placed in a 5 mL centrifuge tube, gently ground. The culture medium was added, filtered through a 200-mesh sieve, and the filtrate was collected with centrifugation at 1000 r/min for 5 min. After discarding the supernatant, red blood cell lysis was added. The solution was shaken and centrifuged. The precipitate was milky white. After washing twice with PBS, the cell concentration was adjusted to 1×10^7^ cells/mL, and 10 μL of CD3-APC, CD4-FITC and CD8a-PE monoclonal antibody were added, respectively. The cells were incubated for 30 min at 4 °C in the dark, and then were detected by flow cytometry.

#### 4.10.5. Detection of Tumor Tissue Lesions by HE Staining

The mice were sacrificed by cervical dislocation and the tumor was stripped and weighed. After weighing, a tumor block of about 5 mm thickness was cut and quickly placed in a 4% paraformaldehyde solution for fixation. The paraffin-embedded blocks were routinely prepared, sliced 5 μm, and routinely dewaxed to water. After conventional HE staining and sealing, the tumor tissue morphology was examined by light microscopy.

#### 4.10.6. Western Blot Analyses

Proteins were extracted from tumor tissue using RIPA (strong) tissue cell lysate for Western blotting. A protein concentration assay kit was applied to measure the protein concentration. The samples were boiled in 5 x SDS loading buffer for 10 minutes and separated by SDS-polyacrylamide gel electrophoresis (PAGE). The isolated protein was transferred to a pure nitrocellulose blotting membrane. These membranes were incubated with 5% (*w*/*v*) skim milk powder dissolved in tris buffered saline containing 0.05% Tween-20 (TBST) for 2 hours at room temperature. Membranes were then incubated with antibodies p53, Bax, BCl-2 and Caspase-3 overnight at 4 °C. After being washed with PBST, the HRP-labeled secondary antibody was incubated with the membrane for 2 hours at room temperature. After re-washing with PBST, the membrane was scanned with a fully automated chemi-luminescence analyzer and the associated strip gray values were read by TANON GIS software.

### 4.11. In Vitro Antitumor Assay

#### 4.11.1. Cell Culture

A549 cells were cultured in DMEM containing 10% fetal bovine serum, 100 g/L penicillin, and 100 g/L streptomycin at 37 °C under 5% CO_2_ saturated humidity until the cells were grown to log phase.

#### 4.11.2. Cell-Growth Inhibition Assay (MTT Assay)

A cell suspension with a concentration of 1 × 10^5^ cells/mL was adjusted in DMEM medium containing 10% FBS and inoculated into a 96-well plate (100 μL per well) at 37 °C in a 5% CO_2_ saturated humidity incubator. The cells were fully adhered for 24 h. The culture solution was aspirated, and the culture medium containing 25, 50, 100, 200, 400, 500, and 1000 μg/mL of AX-I-3b polysaccharide was added. A negative control well was added to 100 μL of serum-free DMEM medium, while the positive control group was added to 10 μg/mL of cisplatin. And six replicate wells per group were set. The culture was continued for 24 h. At the end of the reaction, the culture solution was aspirated. 90 μL of serum-free medium and 10 μL of 5 mg/mL MTT solution were added into each well. The culture was continued for 4 h. After completion of the culture, the liquid was aspirated, 150 μL of prewarmed DMSO at 37 °C was added to each well, and then shaking for 10 min. The absorbance (A) value of the microplate reader was measured at 490 nm, and the cell survival rate was calculated. The same assay was performed on BEAS-2B cells to obtain cytotoxicity results. Cell-proliferation inhibition rate (%) = (1 − A drug/A control group) × 100%.

#### 4.11.3. Cell Cycle and Apoptosis Analysis by Flow Cytometry

A549 cells in the logarithmic growth phase were digested and collected, uniformly inoculated into a six-well plate, and the hemicellulose slag hemicellulose polysaccharide AX-I-3b (0, 200, and 400 μg/mL) was added. A negative control was set. There were three replicates for each concentration. After 24 h, the cells were trypsinized, collected, and washed once with prechilled PBS. Each sample was fixed with 4 °C precooled 70% ethanol for 20 min. Centrifugation (1000 rpm, 5 min) was conducted to remove the fixative. After washing once with PBS, re-suspending in 450 μL of PBS, adding 10 μL of 100 μg/mL RNase and 300 μL of 50 μg/mL PI buffer. Incubation was carried out for 20 min in the dark to protect cells from light. Cell-cycle distribution was measured by flow cytometry (BD, Franklin Lakes, NJ, USA). After treating the cells for 24 h in the same manner as described above, cells were collected, and 5 μL of Annexin V stain and 10 μL of PI stain were added to each sample. The samples were stained for 20 min at room temperature in the dark, and apoptosis rate was detected by flow cytometry. Each experiment was repeated for three times.

### 4.12. Data Processing and Statistical Analysis

All data were statistically analyzed through one-way variance analysis (ANOVA) and t-test using SPSS 16.0 software package (SPSS, Chicago, IL, USA) (company, City, state abbrev if USA, Country). The test level α = 0.05 and *p* < 0.05 were considered statistically significant.

## Figures and Tables

**Figure 3:**
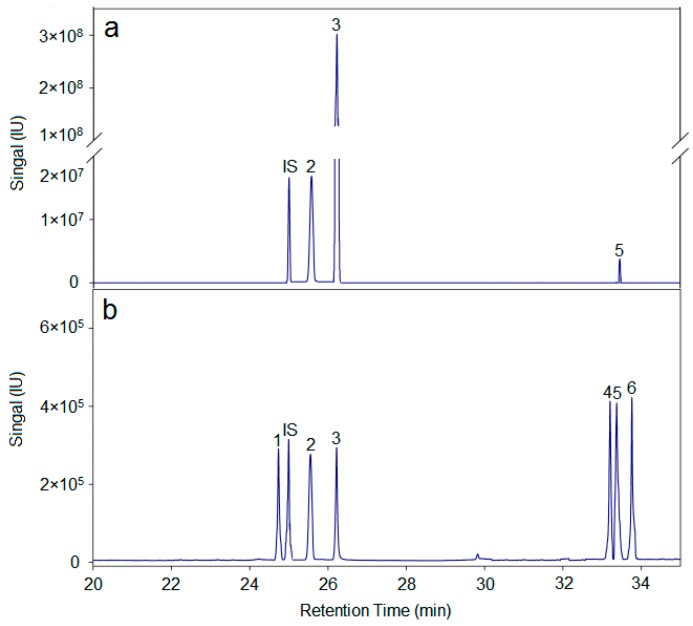
Monosaccharide compositions of AX-I-3b. a is monosaccharide composition analysis of AX-I-3b. (2) arabinose, (3) xylose, (5) glucose; b is a total-ion chromatogram of six monosaccharide controls. (1) rhamnose (t_R_ = 24.73 min); (2) arabinose (t_R_ = 25.55 min); (3) xylose (t_R_ = 26.21 min); (4) mannose (t_R_ = 33.20 min); (5) glucose (t_R_ = 33.37 min); (6) galactose (t_R_ = 33.76 min); IS is the internal standard ribitol (t_R_ = 24.93 min).

**Table 1:** Results of methylation analysis of AX-I-3b.

Peak No.	Methylated	Linkage Types	Molar Ratio
**1**	Xyl-	→2,3,4)-*D*-Xylp-(1→	82.81
**2**	2,3-Me_2_-Ara	→4)-*D*-Arap-(1→	9.23
**3**	2,3,6-Me_4_-Glc	→4)-*D*-Glcp-(1→	1.14

**Table 2:** Chemical shifts of glycosyl ^1^H and ^13^C in AX-I-3b.

Residues	*δ*					
	1-H/C1	2-H/C2	3-H/C3	4-H/C4	5-H/C5	6-H/C6
→4)-*β*-d-Arap-(1→ (A)	4.28/102.0	3.87/74.2	3.66/75.8	3.05/80.2	3.73/63.7	-/-
→2,3,4)-*β*-d-Xylp-(1→ (B)	4.78/102.0	3.05/73.1	3.27/74.5	3.87/75.9	3.42/62.9	-/-
→4)-*β*-d-Glcp-(1→ (C)	5.00/100.6	3.73/71.9	3.87/73.1	4.05/71.9	3.50/74.5	3.73/60.8

## Supplementary Materials

The following are available online at https://www.mdpi.com/1420-3049/24/20/3644/s1.

## References

[1] Wang M.W., Zhou L., Liu S.H., Zhu S. (2017). Discussion on resource utilization of herb residues. J. Guangdong Pharm. Univ..

[2] Pan H.F., Deng Q.D., Feng Y.Z., Zhao Z.M. (2011). Feasibility analysis of comprehensive utilization of traditional Chinese medicine slag. Lishizhen Med. Mater. Med. Res..

[3] Hao X., Li K., Wang G.Z., Liu L., Miao Y.H., Qin X.M. (2016). Extraction, separation and structural analysis of arabinoxylans from the residue of Astragalus root. J. Shanxi Med. Univ..

[4] Cao L., Liu X.Z., Qian T.X., Sun G.B., Guo Y., Chang F.J., Zhou S.M., Sun X.B. (2011). Antitumor and immunomodulatory activity of arabinoxylans: A major constituent of wheat bran. Int. J. Biol. Macromol..

[5] Chaikumpollert O., Methacanon P., Suchiva K. (2004). Structural elucidation of hemicelluloses from Vetiver Grass. Carbohydr. Polym..

[6] Roy A.K., Sen S.K., Bag S.C., Pandey S.N. (1991). Infrared spectra of jute stick and alkali-treated jute stick. J. Appl. Polym. Sci..

[7] Sun R.C., Sun X.F. (2002). Fractional and structural characterization of hemicelluloses isolated by alkali and alkaline peroxide from barley straw. Carbohydr. Polym..

[8] Sun R., Lawther J.M., Banks W.B. (1996). Fractional and structural characterization of wheat straw Hemicelluloses. Carbohydr. Polym..

[9] Pu X.Y., Ma X.L., Liu L., Ren J., Li H.B., Li X.Y., Yu S., Zhang W.J., Fan W.B. (2016). Structural characterization and antioxidant activity in vitro of polysaccharides from angelica and astragalus. Carbohydr. Polym..

[10] Zhou L.J., Liu Z.J., Wang Z.X., Yu S., Long T.T., Zhou X., Bao Y.X. (2017). Immunomodulatory effects of herbal formula of astragalus polysaccharide (APS) and polysaccharopeptide (PSP) in mice with lung cancer. Int. J. Biol. Macromol..

[11] Li W.F., Song K.D., Wang S.P., Zhang C.H., Zhuang M.L., Wang Y.W., Liu T.Q. (2019). Anti-tumor potential of astragalus polysaccharides on breast cancer cell line mediated by macrophage activation. Mater. Sci. Eng. C.

[12] Zhu Z.Y., Liu R.Q., Si C.L., Zhou F., Wang Y.X., Ding L.N., Chen J., Liu A.J., Zhang Y.M. (2011). Structural analysis and anti-tumor activity comparison of polysaccharides from Astragalus. Carbohydr. Polym..

[13] Yu J., Ji H.Y., Dong X.D., Feng Y.Y., Liu A.J. (2019). Apoptosis of human gastric carcinoma MGC-803 cells induced by a novel Astragalus membranaceus polysaccharide via intrinsic mitochondrial pathways. Int. J. Biol. Macromol..

[14] Zhou L.J., Liu Z.J., Wang Z.X., Yu S., Long T.T., Zhou X., Bao Y.X. (2017). Astragalus polysaccharides exerts immunomodulatory effects via TLR4-mediated MyD88-dependent signaling pathway in vitro and in vivo. Sci. Rep..

[15] De C.M.L., Ac D.O.S., Leite E.A., Melo M.M., De C.R.A.F., Cassali G.D., de S.C.M., Souza F.E.M., Caldas I.R., Araújo M.S. (2012). Antitumor effectiveness and toxicity of cisplatin-loaded long-circulating and pH-sensitive liposomes against Ehrlich ascitic tumor. Mon. Not. R. Astron. Soc..

[16] Rodewald H.R. (2008). Thymus organogenesis. Annu. Rev. Immuol..

[17] Panzer S., Madden M., Matsuki K. (1993). Interaction of IL-1 beta, IL-6 and tumour necrosis factor-alpha (TNF-alpha) in human T cells activated by murine antigens. Clin. Exp. Immunol..

[18] Huang X.M., Chen X.Q., Gao G.X., Yu F., Xiao C. (2011). T Follicular Helper Cells in Peripheral Blood of Patients with Immune Thrombocytopenic Purpura. Prog. Mod. Biomed..

[19] Jin C.G., Wang X.C., Wu Z.P., Chen X.Q., Jiang Y.X., Gu Y.L. (2003). The level and significance of T lymphocyte sub-group in peripheral blood in patients with cancer. Cancer Res. Clin..

[20] Lowe S.W., Schmitt E.M., Smith S.W., Osborne B.A., Jacks T. (1993). p53 is required for radiation-induced apoptosis in mouse thymocytes. Nature.

[21] Haunstetter A., Izumo S. (1998). Apoptosis: Basic mechanisms and implications for cardiovascular disease. Circ. Res..

[22] Schmitt C.A., Fridman J.S., Yang M., Baranov E., Hoffman R.M., Lowe S.W. (2002). Dissecting p53 tumor suppressor functions in vivo. Cancer Cell.

[23] Owen-Schaub L.B., Zhang W., Cusack J.C., Angelo L.S., Santee S.M., Fujiwara T., Roth J.A., Deisseroth A.B., Zhang W.W., Kruzel E. (1995). Wild-type human p53 and a temperature-sensitive mutant induce Fas/APO-1 expression. Mol. Cell. Biol..

[24] Müller M., Wilder S., Bannasch D., Israeli D., Lehlbach K., Weber M.L., Friedman S.L., Galle P.R., Stremmel W., Oren M. (1998). p53 Activates the CD95 (APO-1/Fas) Gene in Response to DNA Damage by Anticancer Drugs. J. Exp. Med..

[25] Miyashita T., Reed J.C. (1995). Tumor suppressor p53 is a direct transcriptional activator of the human bax gene. Cell.

[26] Oda1 E., Ohki R., Murasawa H., Nemoto J., Shibue T., Yamashita T., Tokino T., Taniguchi T., Tanaka N. (2000). Noxa, a BH3-Only Member of the Bcl-2 Family and Candidate Mediator of p53-Induced Apoptosis. Science.

[27] Schuler M., Bossy-Wetzel E., Goldstein J.C., Fitzgerald P. (2000). Green DR p53 Induces Apoptosis by Caspase Activation through Mitochondrial Cytochrome c Release. J. Biol. Chem..

[28] Burlacu A. (2003). Regulation of apoptosis by Bcl-2 family proteins. J. Cell. Mol. Med..

[29] Autret A., Martin S.J. (2009). Emerging role for members of the Bcl-2 family in mitochondrial morphogenesis. Mol. Cell.

[30] Gross A., McDonnell J.M., Korsmeyer S.J. (1999). BCL-2 family members and the mitochondria in apoptosis. Genes Dev..

[31] Acehan D., Jiang X., Morgan D.G., Heuser J.E., Wang X., Akey C.W. (2002). Tree-dimensional structure of the apoptosome: Implications for assembly, procaspase-9 binding, and activation. Mol. Cell.

[32] Pradelli L.A., Beneteau M., Ricci J.E. (2010). Mitochondrial control of caspase-dependent and -independent cell death. Cell. Mol. Life Sci..

[33] Yakovlev A.G., Wang G., Stoica B.A., Boulares H.A., Spoonde A.Y., Yoshihara K., Smulson M.E. (2000). A role of the Ca^2+^/Mg^2+^-dependent endonuclease in apoptosis and its inhibition by Poly(ADP-ribose) polymerase. J. Biol. Chem..

[34] Pearce A., Haas M., Viney R., Pearson S.A., Haywood P., Brown C., Ward R. (2017). Incidence and severity of self-reported chemotherapy side effects in routine care: A prospective cohort study. PLoS ONE.

[35] Naidu M.U., Ramana G.V., Rani P.U., Mohan I.K., Suman A., Roy P. (2004). Chemotherapy-induced and/or radiation therapy-induced oral mucositis-Complicating the treatment of cancer. Neoplasia.

[36] Hayakawa Y., Smyth M.J. (2006). Innate Immune Recognition and Suppression of Tumors. Adv. Cancer Res..

[37] Wu H., Wei J.H., Zheng J., Ren Q.Y. (2017). Discussion on relationship between yang deficiency and tumor formation. China J. Tradit. Chin. Med. Pharm..

[38] Liang Y., Huang Y. (2018). Advances in tumor-associated macrophages involved in tumor immunoregulation. Prog. Anat. Sci..

[39] Li J.Y., Zhang F.C. (2016). Research Progress on Anti-tumor Immune Regulation Mechanism of Traditional Chinese Medicine. Lishizhen Med. Mater. Med. Res..

[40] Hori S., Nomura T., Sakaguchi S. (2003). Control of Regulatory T Cell Development by the Transcription Factor Foxp3. Science.

[41] Curiel T.J., Coukos G., Zou L., Alvarez X., Cheng P., Mottram P., Evdemon-Hogan M., Conejo-Garcia J.R., Zhang L., Burow M. (2004). Specific recruitment of regulatory T cells in ovarian carcinoma fosters immune privilege and predicts reduced survival. Nat. Med..

[42] Yuan H., Zhang S.F., Jia S.H., Chen N. (2014). Progress in Application and Biological Activity of Radix Astragali in Health Foods. Food Sci..

[43] Zhou Y., Li S., Qian Q., Zeng D., Zhang M., Guo L., Liu X., Zhang B., Deng L., Liu X. (2009). BC10, a DUF266-containing and Golgi-located type II membrane protein, is required for cell-wall biosynthesis in rice (*Oryza sativa* L.). Plant J..

[44] Sun J.X., Sun X.F., Sun R.C., Su Y.Q. (2004). Fractional extraction and structural characterization of sugarcane bagasse hemicelluloses. Carbohydr. Polym..

[45] Cheng X.Q., Li K., Chen X.M., Jiang X.N., Gai Y. (2012). Comparison of pectin structural monosaccharides in cell wall of dicotyledon and monocotyledon. J. Beijing Univ..

[46] Zha S.H., Zhao Q.S., Chen J.J., Wang L.W., Zhang G.F., Zhang H., Zhao B. (2014). Extraction, purification and antioxidant activities of the polysaccharides from maca (Lepidium meyenii). Carbohydr. Polym..

[47] Liu Y., Yin L., Gong G.P., Peng Y.F., Huang L.J., Wang Z.F. (2016). Structural Characterization, Antioxidant Activity and Immunomodulatory Activity of the Polysaccharide LRLP3 from Leaves of Lycium ruthenicum Murra†. Chem. J. Chin. Univ..

[48] Xia C.H., Dai Q., Fang W., Chen H.S. (2007). Research on the IR Spectroscopy of Kinds of Polysaccharide. J. Wuhan Univ. Technol..

[49] Gao F.R., Li K., Hao X., Wang G.Z., Qin X.M. (2015). Identification of cultured and natural Astragali Radix based on fingerprint of monosaccharides. Chin. Tradit. Herb. Drugs.

[50] Prozil S.O., Costa E.V., Evtuguin D.V., Lopes L.P., Domingues M.R. (2012). Structural characterization of polysaccharides isolated from grape stalks of *Vitis vinifera* L.. Carbohydr. Res..

[51] Košťálová Z., Hromádková Z. (2019). Structural characterisation of polysaccharides from roasted hazelnut skins. Food Chem..

[52] Yang B., Xiao B., Sun T. (2013). Antitumor and immunomodulatory activity of Astragalus membranaceus polysaccharides in H22 tumor-bearing mice. Int. J. Biol. Macromol..

[53] Zheng Y., Wang W.D., Li Y. (2015). Antitumor and immunomodulatory activity of polysaccharide isolated from Trametes orientalis. Carbohydr. Polym..

