# Supplementary material for: Extraction, Characterization, Antitumor and Immunological Activities of Hemicellulose Polysaccharide from Astragalus radix Herb Residue
Source: Molecules. 2019 Oct 9;24(20):3644. doi: 10.3390/molecules24203644 (PMC6833037; doi:10.3390/molecules24203644)
Supplement: Supplementary file 1 [file molecules-24-03644-s001.pdf]

Supplementary data

Fig. S1. Standard curve of the retention time of dextran standard reference and the logarithm of its corresponding molecular weight

Fig. S2. Effect of AX-I-3b on the A549 cell cycle (24 h)

Fig. S3. Apoptosis rate of AX-I-3 on A549 cells for 24 h (***P < 0.001 compared with the negative control group)

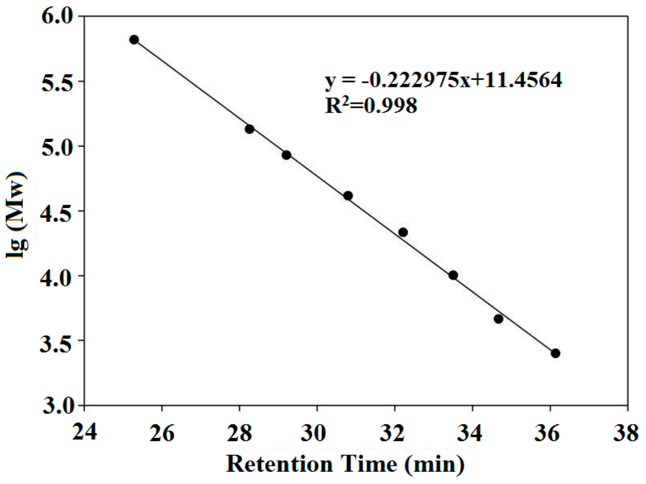

Fig. S1. Standard curve of the retention time of dextran standard reference and the logarithm of its corresponding molecular weight

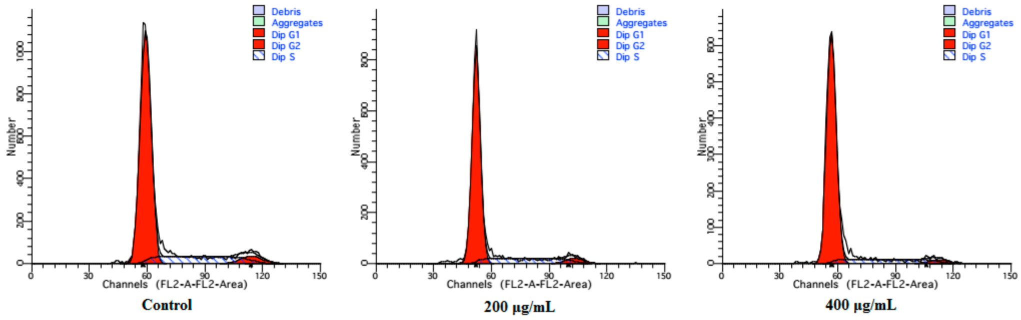

Fig. S2. Effect of AX-I-3b on the A549 cell cycle (24 h)

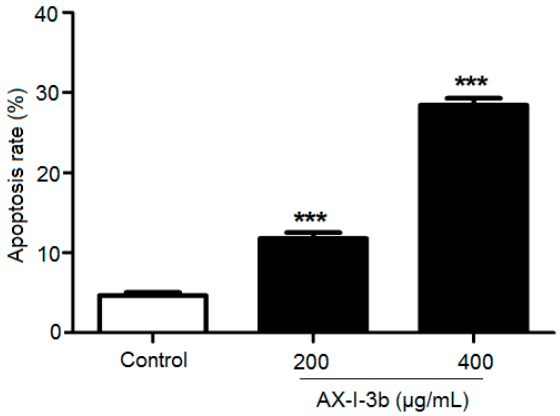

Fig. S3. Apoptosis rate of AX-I-3 on A549 cells for 24 h (***P < 0.001 compared with the negative control group)

Table S1

Retention time of dextran standard reference and the logarithm of its corresponding molecular weight

Table S2

Results of monosaccharide composition analysis of AX-I-3

Table S3

Effect of AX-I-3 on the A549 cell cycle (24 h)

Table S1

Retention time of dextran standard reference and the logarithm of its corresponding molecular weight.

	Relative molecular mass (Mw)							
	2500	4600	10000	21400	41100	84400	133800	655200 (glucan)
lg (Mw)	3.398	3.663	4.000	4.330	4.614	4.926	5.126	5.816
t_R (min)	36.149	34.682	33.515	32.223	30.807	29.215	28.265	25.294

Table S2

Results of monosaccharide composition analysis of AX-I-3

	Monosaccharide composition (molar ratio)					
	Rha	Ara	Xyl	Man	Glc	Gal
AX-I-3	-	10.4	79.3	-	1.1	-

Table S3

Effect of AX-I-3 on the A549 cell cycle (24 h)

Group	G0/G1	S	G2/M

Control	77.03 ± 0.5	17.96 ± 0.7	5.01 ± 0.2
AX-I-3 (200 μg/mL)	80.44 ± 0.9*	15.7 ± 0.5*	3.85 ± 0.1*
AX-I-3 (400 μg/mL)	84.17 ± 0.7**	13.34 ± 0.5**	2.49 ± 0.1**
